# Sublaminar stabilization (stapling) of the caudal cervical instability in Yorkshire terrier—A case report

**DOI:** 10.3389/fvets.2025.1606037

**Published:** 2025-07-02

**Authors:** Jacek Sterna, Beata Degórska, Joanna Bonecka, Mikhal Baranski

**Affiliations:** Department of Small Animal Diseases and Clinic, Institute of Veterinary Medicine, Warsaw University of Life Sciences, Warsaw, Poland

**Keywords:** sublaminar stabilization, caudal cervical instability, miniature dog, Yorkshire terrier, ataxia

## Abstract

This case report describes the long-term outcome of surgical treatment of rare spinal instability in small-breed dogs. A 2-year and 7-month-old male Yorkshire terrier, weighing 1.5 kg, was presented with tetraparesis and proprioceptive deficits, neck pain, and intact deep pain sensation for a clinical examination. The conservative treatment had been unsuccessful for 20 months. A diagnosis of C6-C7 instability with a slight vertebral subluxation was made, which led to the decision about sublaminar stabilization. The Kirschner wire was placed on both sides of the spinous processes from C5 to T1 using the left dorsal approach. A polyamide monofilament suture was passed under the laminas of the cervical fifth, sixth, and seventh vertebras and tied around the Kirschner wire. The cranial end of the Kirschner wire had been stuck under the lamina of the fourth cervical vertebra after the surgery. A reoperation was performed 33 days later. The cranial end of the Kirschner wire was removed from under the lamina and bent dorsally. The dog was ambulatory and without neck pain 2 days after the reoperation. Five years after the surgical treatment, the dog is still ambulatory with normal proprioceptive positioning but with subtle ataxia. This kind of stapling sublaminar stabilization may be a useful method as a primary means for stabilization in cases of cervical spine instability in small dogs. It may also serve as a secondary procedure in cases of severe complications after the failure of other methods applied from the ventral approach.

## Introduction

The cervical part of the vertebral column consists of seven vertebras and is the most movable part of the spine. The damage to the cervical spinal cord in small and miniature breed dogs, besides the atlantoaxial instability, is usually of traumatic origin, unlike in large breed dogs where spondylomyelopathy in the caudal part of the neck spine is common (1).

In cases of cervical spinal cord damage requiring stabilization of vertebras, a variety of methods can be applied, from external coaptation in toy-breed dogs (2) through articular facets wiring (3), to locking compression plates (4), transpedicular screws and polymethyl methacrylate applied with the help of patient-specific 3-dimensional (3D) printed drill guide (5).

In small animals' thoracolumbar and lumbar spine, a segmental fixation with modifications, also called a spinal stapling, is beneficial (6). A similar technique has also recently been used in humans (7).

The treatment of the cervical spine in dogs by the sublaminar stabilization is limited to the atlantoaxial instability. It is subject to a more considerable risk of failure than the surgical stabilization from the ventral approaches (8). Sublaminar stabilization is broadly used in human patients, especially in cases of spinal deformities, where mainly tapes are used for the connection of the vertebral arches and longitudinal bars (9), but also in the treatment of fractures (10, 11). There are no reports about the surgical treatment of the instability in the caudal part of the cervical spine by a stapling modified by sublaminar application of monofilament threads in miniature dogs.

## Patient information

A 2-year and 7-month-old male Yorkshire terrier weighing 1.5 kg was presented at the Small Animal Clinic of the Faculty of Veterinary Medicine of the Warsaw University of Life Sciences. According to the medical history, the dog had fallen off a balcony at the second floor 20 months before. The dog's mobility difficulties were initially diagnosed as typical luxation of the right patella, and later as instability between C6 and C7. The diagnosis had been confirmed by the magnetic resonance imaging (MRI) scan performed in another clinic a few days later. The scan indicated a C6-C7 disc/nucleus pulposus injury and instability/subluxation. The conservative treatment (nonsteroidal anti-inflammatory drugs, as well as cage rest and neck and chest brace) had been unsuccessful. In this case, it meant that 2–3 weeks after the cage or corset restriction had ended, the symptoms of tetraparesis and neck pain returned.

## Clinical findings

During the presentation at the Small Animal Clinic, the dog was tetraparetic, with delayed paw position responses, which were more delayed in the left limbs and neck pain symptoms.

## Diagnostic assessment

**Figure 1:**
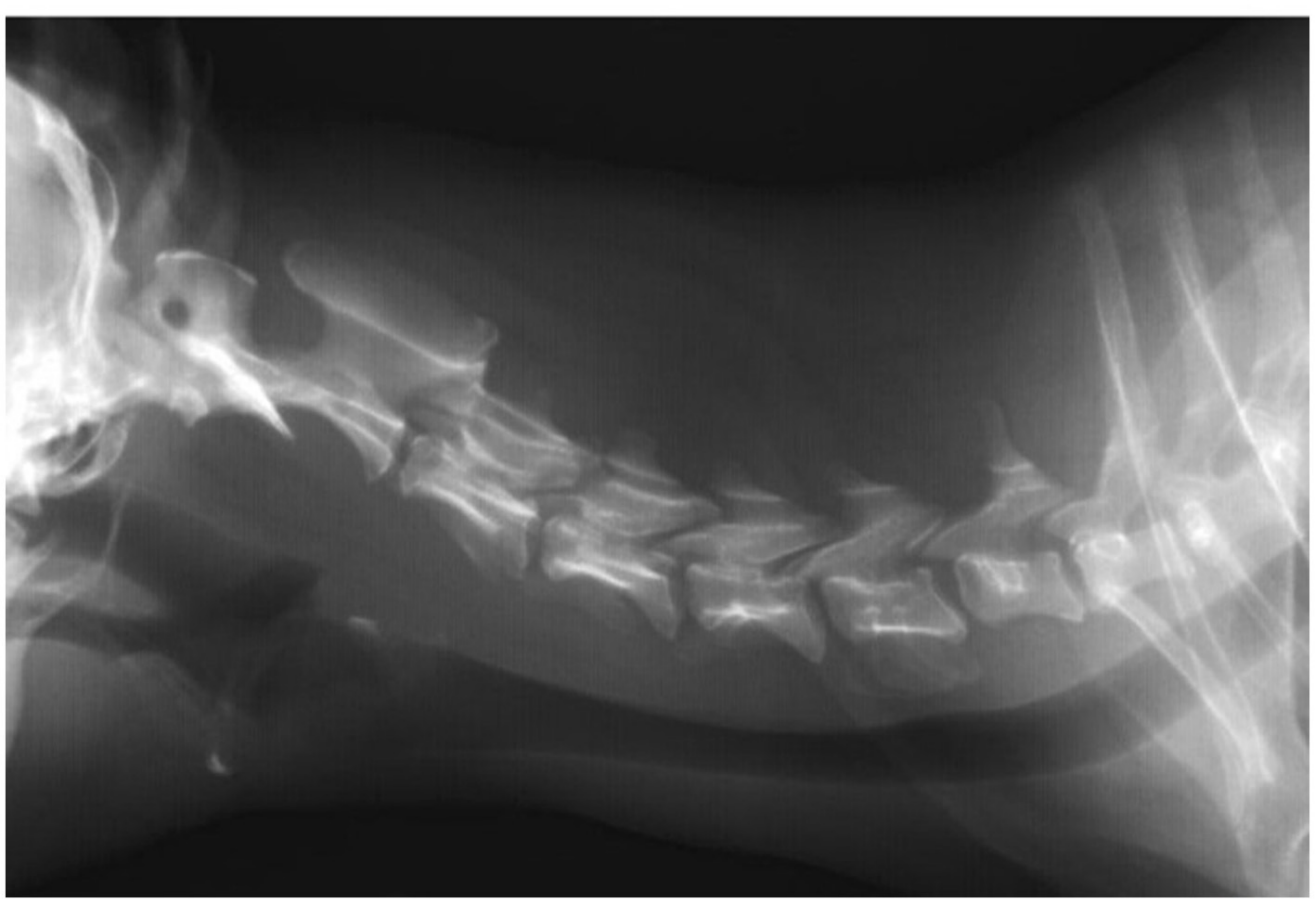
Radiographic image taken before surgery. A lateral plain radiograph of the cervical vertebral column in neutral. The radiograph reveals severe C6-7 instability/subluxation.

Diagnostic methods included blood tests, clinical and neurological examinations, MRI images from previous clinics, and dynamic radiographs. Blood tests showed slightly elevated levels of BUN (65,50 mg/dl) and alanine aminotransferase (62,50 U/l). Based on the examination results, C6-C7 instability and slight vertebral subluxation were diagnosed (Figure 1). Due to the size of the patient and thin and fragile vertebral bodies, surgical stapling was decided. The treatment prognosis was guarded due to the chronicity of instability, patient size, and thickness of bone structures.

## Therapeutic intervention

**Figure 2:**
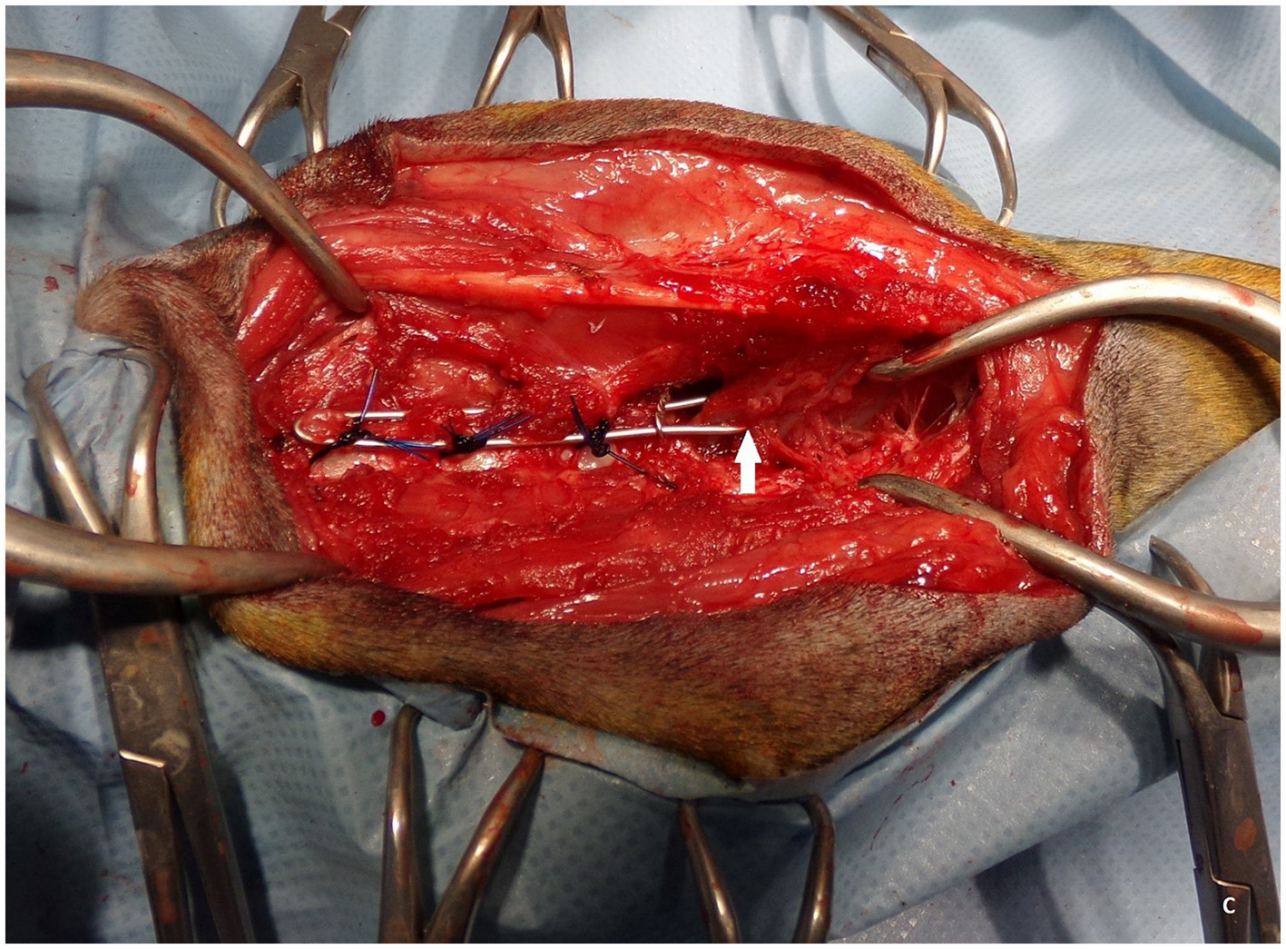
The staple attached to the vertebral arches of C5, C6, and C7 with blue monofilament thread. The cerclage is visible to the right from C7, compressing both arms of the staple between C7 and Th1. The white arrow shows at which point the left arm of the staple was bent behind the spinous process of Th1. The head of the dog is to the left of the picture.

The owner accepted the surgical treatment proposal, i.e., a stabilization procedure. On the day of surgery, the dog was premedicated intramuscularly with dexmedetomidine (5 μg/kg; Dexdomitor, Orion Pharma) and butorphanol (0.2 mg/kg; Torbugesic, Zoetis). Anesthesia was induced with propofol (1 mg/kg; Propofol-Lipuro, B. Braun Melsungen), and maintained with isoflurane (Iso-Vet, Piramal) with a constant rate infusion of fentanyl (10 μg/kg/h initially, dose adjusted based on the patient's vital parameters; fentanyl WZF, Polfa Warszawa). The dog was prepared for surgery *lege artis*, and placed in sternal recumbency. The dorsal approach to the caudal cervical and cranial thoracic vertebrae limited to the left side was performed. The spinous processes of the C4-5-6-7-T1-2-3 vertebrae and their arches on the left side were exposed. Sections of polyamide monofilament 3 (Amifil M Sinpo), about 30 cm each, two for one vertebral arch, were passed under the arches of the C5, C6, and C7 vertebrae on the left side from the spinous processes by using a cerclage wire loop. A section of the Kirschner wire, about 15 cm long and 1 mm thick, was bent in the middle of its length and curved to mimic the normal lordosis of this part of the spine, according to the shape visible on the latero-lateral radiographic view taken without bending of the spine. The wire was adjusted to fairly closely encompass the spinous processes of the C5, C6, and C7 vertebrae; about 6 mm of its end on the left side was bent just behind the spinous process of T1. The right end of the Kirschner wire ended on the right side of the spinous process of T3. The bent Kirschner wire was similar to a “staple” (6) and will be referred to as such in what follows. Double monofilament threads were tied with surgical knots around the left part of the staple (Figure 2). A cerclage was placed between the spinous processes of the C7 and T1 vertebrae on the staple, and another cerclage was placed encompassing the staple and the spinous process of T1. The wound was closed in layers. Two orthogonal radiographs were made. The postoperative analgesia consisted of the administration of meloxicam (0.2 mg/kg as the first dose, then 0.1 mg/kg), metamizole (25 mg/kg every 8 h), and buprenorphine (2 μg/kg every 6 h intramuscularly).

The dog was hospitalized for 24 h. At the time of discharge, the dog had paresis but no symptoms of neck pain. Amoxicillin with clavulanic acid (14 mg/kg SC) once a day (Synulox, Haupt Pharma Latina) for 5 days, and a cervicothoracic corset (brace) was prescribed for 3 weeks. A limited movement of the dog on a short leash was recommended. A follow-up examination was scheduled 1 month after surgery, due to owner's, who lived far from the clinic.

**Figure 3:**
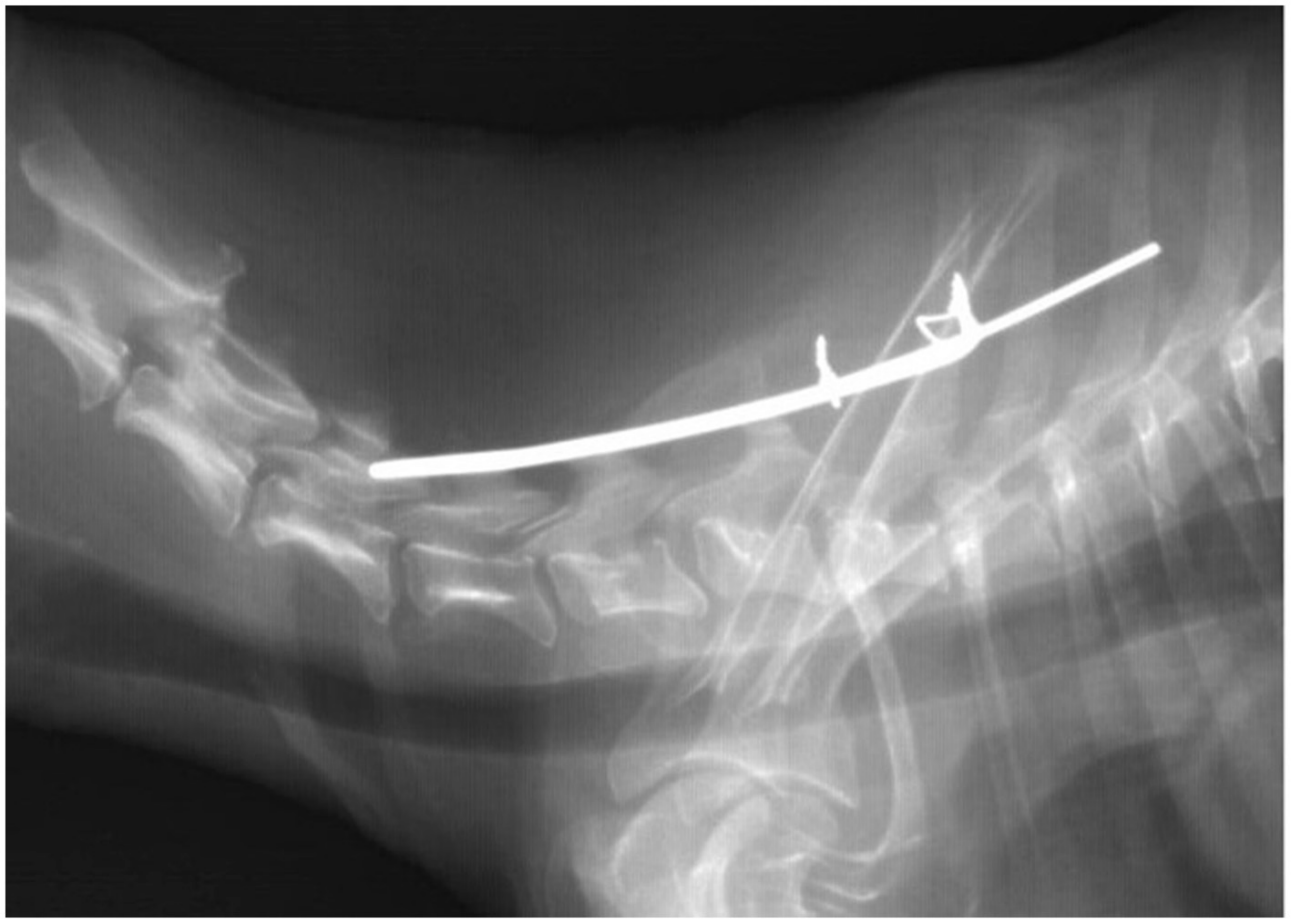
Radiographic image taken four weeks after the first surgery. Dislocated the cranial end of the Kirschner wire (a staple) is visible.

During the first month after surgery, the dog, according to telephone information from the referred veterinarian and the owner, was ambulatory but had difficulty standing up and often vocalized pain. A follow-up examination in the Clinic revealed paresis of the thoracic limbs, no tactile placement, and neck pain symptoms. A radiologic examination was performed under the same premedication used on the surgery day to facilitate calm and effective imaging studies. It turned out that cranial end of the staple had migrated under the arch of the C4 vertebra (Figure 3), which was considered an indication for re-operation.

Three days later, the second surgery was performed, using the same anesthesiology protocol and the same surgical approach as described previously. After slight ventral flexing of the cranial part of the neck, the end of the staple was freed from under the arch of the C4 vertebra. The caudal edge of the C4 arch and the cranial edge of the C5 arch were slightly worn down due to rubbing against the staple. That end of the staple was bent in the dorsal direction with the help of flat-nose pliers. In addition, the torn monofilament surrounding the C5 vertebral arch and the staple was removed. An attempt was made to place another monofilament, but the staple and tissue adhesion around the vertebral arch made it difficult. As the C6-C7 instability was the main issue, we abandoned further manipulation in this area. The wound was closed routinely. Post-operative radiography showed the correct placement of the staple. All the postsurgical procedures were the same as after the first surgery.

## Follow-up and outcomes

According to the dog's owner's telephone report, the difficulties with standing up and the signs of pain disappeared 2 days after reoperation. Ten days after the re-operation, at the follow-up examination, the owner claimed that the difficulty in standing up and pain symptoms disappeared and that the dog was running willingly and standing without difficulty on three limbs when urinating. During the examination in the Clinic, the dog was ambulatory, still ataxic, with delayed tactile placing, had slightly increased four limb reflexes, and no pain. The cervicothoracic corset was prescribed for the next 2 weeks to reduce the dog‘s activity. Six weeks after the re-operation, the dog was ambulatory (and moved willingly) with slight ataxia of the pelvic limbs, but placement reactions were normal. No pain symptoms were noted.

**Figure 4:**
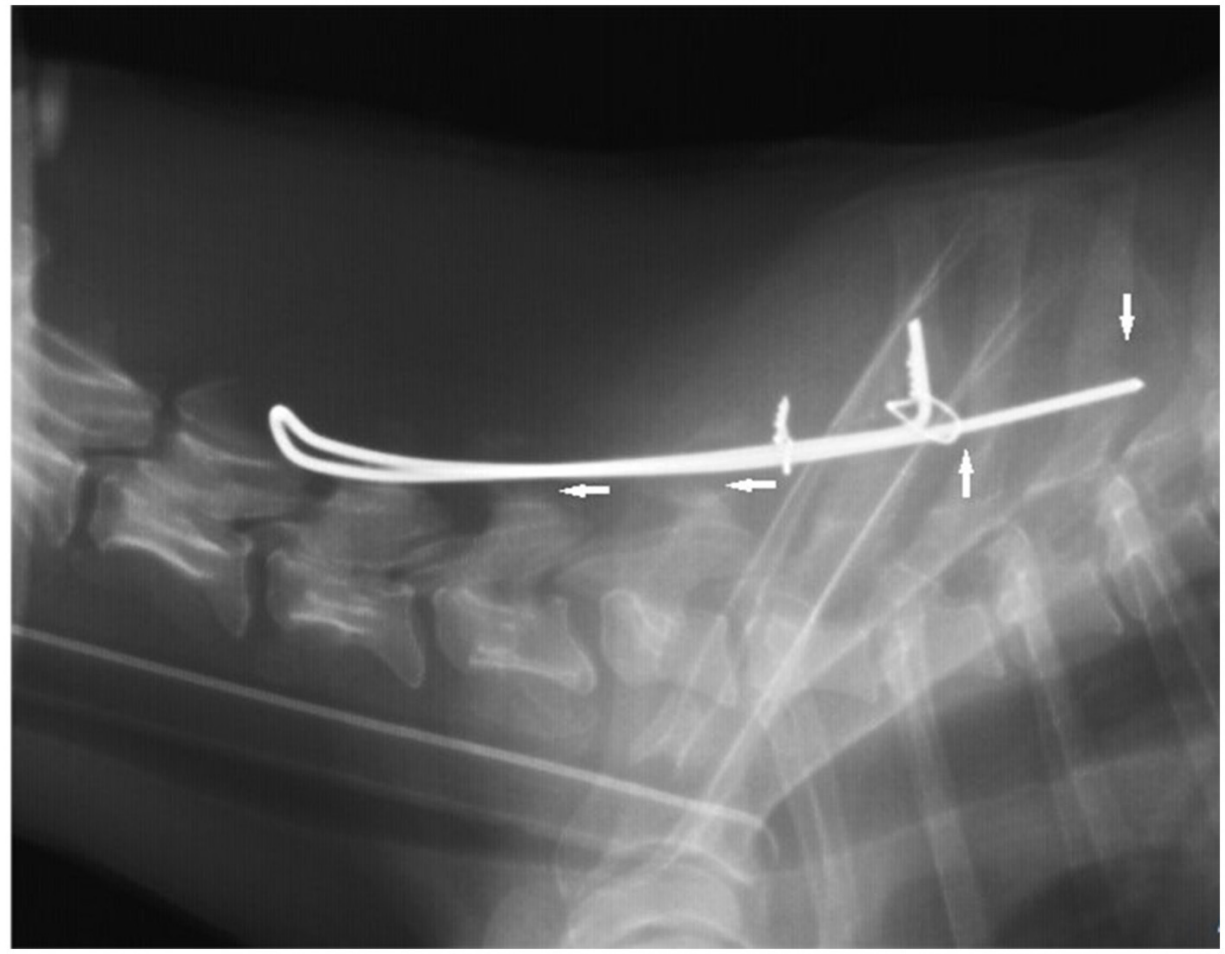
The radiographic image was taken 6 months after the first surgery. Note a gap of about 1.5 mm over the C6 arch and about 3.5 mm over the C7 arch (horizontal arrows) and osteolysis (vertical arrows) where the staple adhered to the spinous processes of T1 and T3 on the lateral view.

Six months after the re-operation, the dog was ambulatory, without ataxia, and had normal proprioception. No pain symptoms were noted. A radiographic examination was performed under sedation (as previously). The staple had slightly moved dorsally, with a gap of about 1.5 mm over the C6 arch and about 3.5 mm over the C7 arch. There were also features of osteolysis where the staple adhered to the spinous processes of T1 and T3 (Figure 4).

At a follow-up examination approximately 12 months after the re-operation, the dog was ambulatory (walked without any non-compliance) but with a slightly delayed placement reaction in the left limbs. No pain symptoms were noted. An X-ray examination was performed under sedation (as previously). The left part of the staple was broken over the C6, and gaps were visible between the vertebral arches of the C5, C6, C7, and the staple. Features of osteolysis were still visible as in the previous examination.

At a follow-up examination about 5 years after the re-operation, the dog was ambulatory, walked with subtle ataxia. Paw position response and tactile placing tests were normal in the four limbs. Reflexes in the front limb were slightly weakened. The dog flexed the left front limb while lying down, but the posture during urination was normal, despite second-grade medial patellar luxation in the right pelvic limb. On an X-ray image, the gap between the C7 vertebral arch and the staple and the signs of vertebral osteolysis had decreased compared to the previous examination; additionally, the right part of the staple was broken, but without any influence on the animal's condition.

## Discussion

According to our knowledge, stapling with sublaminar sutures in the caudal cervical spine in a dog with spinal cord damage was not previously considered. In cervical vertebral fractures or instability cases, a combination of metallic implants and bone cement is predominantly used (2, 12). The combination can be easily applied to dogs and cats of any size and weight, especially small ones.

The anatomy of the cervical vertebrae of miniature dogs makes it relatively easy to run a thread or tape under the vertebral arch because of the fairly wide gaps between the vertebral arches. Unlike the use of numerous metallic implants and bone cement or screws and plates, the method used in this report avoids drilling into the direction of the spinal cord. The monofilament is carried out under the vertebral arches using a thin cerclage wire in a direction tangential to the dura. In some cases, sublaminar stabilization (stapling) may avoid a two-stage surgery (repositioning from the dorsal approach and stabilization from the ventral side) (2).

Even though the staple broke and probably the monofilament was torn, and gaps between the staple and the vertebral arches appeared, the dog was still moving very efficiently and was considered by its owner as healthy. Long-term radiological examination showed a slight dorsal dislocation of the C7, but no visible abnormalities. Soft tissue stabilization was sufficient to protect the vertebra against excessive movement that might result in pain or mobility difficulties.

This is only one case that we treated using the presented method. As described above, some complications were observed (stacking of the cranial end of the staple under the vertebral lamina, break of the staple, a torn or loosening of the monofilament, and some foci of osteolysis). This first problem was important but solved by simple dorsal bending of the cranial end of the staple. The rest of the complications probably did not adversely affect the patient. It is possible that different materials (a polymeric bar instead of the Kirschner wire and braided tape instead of monofilament) could be more durable/suitable and avoid the complications mentioned above. For instance, instead of the Kirschner wire, Whitehill et al. (13) reported the use of U-shaped Steinman pins or bars (struts) made of bone (autologous ulna) after partial laminectomy in the cervical part (C4-C5) in experimental dogs weighing 15–20 kg.

Instead of a cerclage wire, various tapes are used during the surgery in the sublaminar stabilization in children with scoliosis (10, 14, 15).

## Conclusions

We believe that the sublaminar stapling may be a valuable method for stabilization in cases of cervical spine instability in small dogs. It may also serve as a secondary procedure in cases of severe complications after the failure of other methods applied from the ventral approach.

## Data Availability

The datasets presented in this study can be found in online repositories. The names of the repository/repositories and accession number(s) can be found in the article/supplementary material.

## References

[1] Platt SR Da Costa RC. Cervical vertebral column and spinal cor. In:Johnston S Tobias K, editors. Veterinary Surgery Small Animal, vol. 1, 2nd ed. Saint Louis, MO: Elsevier (2018). p. 438–85

[2] Woelfel CW Bray KY Early PJ Mariani CL Olby NJ Subaxial cervical articular process subluxation and dislocation: cervical locked facet injuries in dogs. Vet Surg. (2022) 51:163–172. 10.1111/vsu.13746 34820884

[3] Basinger RR Bjorling DE Chambers JN. Cervical spinal luxation in two dogs with entrapment of the cranial articular process of C6 over the caudal articular process of C5. Am Vet Med Assoc. (1986) 188:865–67. 10.2460/javma.1986.188.08.865 3710877

[4] Trotter EJ. Cervical spine locking plate fixation for treatment of cervical spondylotic myelopathy in large breed dogs. Vet Surg. (2009) 38:705–18. 10.1111/j.1532-950X.2009.00541.x 19674414

[5] Hamilton-Bennett SE Oxley B Behr S. Accuracy of a patient-specific 3D printed drill guide for placement of cervical transpedicular screws. Vet Surg. (2018) 47:236–42. 10.1111/vsu.12734 29064584

[6] Voss K Montavon PM. Tension band stabilization of fractures and luxation of the thoracolumbar vertebrae in dogs and cats: 38 cases (1993–2002). J Am Vet Med Assoc. (2004) 225:78–83. 10.2460/javma.2004.225.78 15239477

[7] Adeolu AA Komolafe EO. Outcome of a posterior spinal fusion technique using spinous process wire and vertical strut. Ann Afr Med. (2014) 13:30–4. 10.4103/1596-3519.126947 24521576

[8] Denny HR Gibbs C Waterman A. Atlantoaxial subluxation in the dog: a review of thirty cases and an evaluation of treatment by lag screw fixation. J Small Anim Pract. (1988) 26:37–47. 10.1111/j.1748-5827.1988.tb02262.x

[9] Mazda K Ilharreborde B Even J Lefevre Y Fitoussi F Pennecot G-F. Efficacy and safety of posteromedial translation for correction of thoracic curves in adolescent idiopathic scoliosis using a new connection to the spine: the Universal Clamp. Eur Spine J. (2009) 18:158–69. 10.1007/s00586-008-0839-y 19089466 PMC2899338

[10] Patil SS Bhojaraj SY Nene AM. Safety and efficacy of spinal loop rectangle and sublaminar wires for osteoporotic vertebral compression fracture fixation. Asian J Neurosurg. (2017) 12:436–40. 10.4103/1793-5482.175648 28761521 PMC5532928

[11] Unterweger M-T Kandziora F Schnake KJ. Hybrid stabilization of thoracic spine fractures with sublaminar bands and transpedicular screws: description of a surgical alternative and review of the literature. Case Rep Orthop. (2015) 2015:857607. 10.1155/2015/857607 26649214 PMC4663301

[12] Fernandes R Fitzpatrick N Rusbridge C Driver CJ. Cervical vertebral malformations in 9 dogs: radiological findings, treatment options and outcomes. Ir Vet J. (2019) 72:2. 10.1186/s13620-019-0141-9 31044069 PMC6480486

[13] Whitehill R Wilhelm CE Moskal JT Kramer SJ Ruch WW. Posterior strut fusion to enhance immediate postoperative cervical stability. Spine. (1986) 11:6–13. 10.1097/00007632-198601000-00003 3704785

[14] Montgomery BK Nandyala SV Birch CM Hogue G. Double sublaminal band passage technique for spinal deformity correction. Cureus. (2022) 14:e22719. 10.7759/cureus.22719 35371806 PMC8971098

[15] Bogie R Arts JJ Koole SN Van Rhijn LW Willems PC. The use of metal sublaminar wires in modern growth guidance scoliosis surgery: a report of 4 cases and literature review. Int J Spine Surg. (2020) 14:182–88. 10.14444/7017 32355624 PMC7188087

